# Dataset of fitness trackers and smartwatches to measuring physical activity in research

**DOI:** 10.1186/s13104-022-06146-5

**Published:** 2022-07-16

**Authors:** André Henriksen, Ashenafi Zebene Woldaregay, Miroslav Muzny, Gunnar Hartvigsen, Laila Arnesdatter Hopstock, Sameline Grimsgaard

**Affiliations:** 1 Department of Community Medicine, UiT The Arctic University of Norway, Tromsø, Norway; 2 Department of Computer Science, UiT The Arctic University of Norway, Tromsø, Norway; 3 Norwegian Centre for E-Health Research, University Hospital of North Norway, Tromsø, Norway

**Keywords:** Motor activity, Activity tracker, Smart watch, Heart rate, Photoplethysmography, Wearables

## Abstract

**Objectives:**

Accelerometer-based wrist-worn fitness trackers and smartwatches (wearables) appeared on the consumer market in 2011. Many wearable devices have been released since. The objective of this data paper is to describe a dataset of 423 wearables released before July 2017.

**Data description:**

We identified wearables and extracted information from six online and offline databases. We also visited websites for all identified companies/brands to identify additional wearables, as well as obtained additional information for each identified device. Twelve attributes were collected: wearable name, company/brand name, release year, country of origin, whether the wearable was crowd funded, form factor (fitness tracker or smartwatch), and sensors supported. Support for the following sensors were mapped: accelerometer, magnetometer, gyroscope, altimeter or barometer, global-positioning-system, and optical pulse sensor (i.e., photoplethysmograph). The search was conducted between May 15th and July 1st, 2017. The included data gives an overview of most in-scope wearables released before July 2017 and allows researchers to conduct additional analysis not performed in the related article. Further insights can be achieved by complementing this list with wearable models released after July 2017.

## Objective

Accelerometer-based wrist-worn fitness trackers and smartwatches (wearables) appeared on the consumer market in 2011 [1]. Many wearable devices have been released since. In addition to being used for personal physical activity tracking, these wearables are also increasingly being used in research as tools to collect health data [2–4]. These wearables are especially designed for long-term usage and thus facilitates long-term recording with low participant burden.

The objective of this data paper is to describe a dataset containing most in-scope wearables (n = 423) released before July 2017. In a study published in 2018, where this data set was used, we showed how sensor support changed over time, and that (in addition to accelerometer) optical pulse sensor (i.e., photoplethysmograph [PPG]) and global positioning system (GPS) were the two most common sensors [1].

## Data description

The dataset contains information on 423 wrist-worn consumer-based wearables, produced by 132 different companies. The dataset is stored at DataverseNO [5]. A ReadMe-file (Table 1, Data file 1) describes the content of the dataset. The data are stored in two files, containing the same information using different formats, where “data.csv” (Table 1, Dataset 1) is a comma-separated file and “data.txt” (Table 1, dataset 2) is a tab-separated file. See Table 1.

**Table 1:** Overview of data files/data sets

Label	Name of data file/data set	File type (file extension)	Data repository and identifier (DOI or accession number)
Data file 1	00_ReadMe.txt	Plain text file (.txt)	DataverseNO (http://doi.org/10.18710/6ZWC9Z*)* [5]
Data set 1	data.csv	Comma separated values (.csv)	DataverseNO (http://doi.org/10.18710/6ZWC9Z*)* [5]
Data set 2	data.txt	Tab separated values (.txt)	DataverseNO (http://doi.org/10.18710/6ZWC9Z*)* [5]

Data were collected in two steps. In step one, we identified wearables and extracted information from six online and offline databases: The Queen’s University’s Wearable Device Inventory [6, 7], The Vandrico Wearables database [8], GsmArena.com [9], Wearables.com [10], SpecBucket.com [11], and PrisGuide.no [12]. In step two, we visited websites for all companies/brands identified in step one to identify additional wearables and extract additional information for each identified wearable.

Conflicting information from the databases was resolved by visiting brand websites. If no website could be found, or the website did not contain the needed information, we used Google search and other online resources (e.g., Wikipedia).

We collected 12 variables for each wearable. Six variables were related to sensor support and six variables were background attributes (i.e., meta data). The six sensor variables are: (1) accelerometer, (2) magnetometer, (3) gyroscope, (4) altimeter or barometer, (5) GPS, and (6) PPG.

The six background attributes are: (1) wearable name, (2) company/brand name, (3) release year, (4) country of origin, (5) whether the wearable was crowd funded, and (6) form factor (fitness tracker or smartwatch). An overview of each variable with a short description is available in the 00_ReadMe-file (Table 1, Data file 1).

The search was conducted between May 15th and July 1st, 2017. Inclusion criteria were: (1) wrist-worn, (2) consumer-based, (3) equipped with accelerometer for physical activity monitoring, and (4) capable of transferring data to connected smartphone.

## Limitations

Despite a thorough search of wearables, the list is likely not complete. During the search we only used English or Norwegian sources, or sources that could be translated using Google Translate. It is therefore possible that among the wearables we did not identify, a predominance of non-western countries is represented. We used multiple sources to map wearable capabilities, sometimes with conflicting information. We visited official vendor websites to identify the correct information, but these were not always available, and some misclassifications may thus exist.

The data set contains variables released between 2011 and 2017, as the search was conducted in 2017. We invite other researchers to update this list with newer models released after 2017, using this data set as a starting point.

## Data Availability

The data described in this Data note can be freely and openly accessed on DataverseNO under https://doi.org/10.18710/6ZWC9Z. Please see Table 1 and references [5] for details and links to the data.

## Abbreviations

PPG: Photoplethysmograph
GPS: Global positioning system

## References

[1] Henriksen A, Haugen Mikalsen M, Woldaregay AZ, Muzny M, Hartvigsen G, Hopstock LA, Grimsgaard S (2018). Using fitness trackers and smartwatches to measure physical activity in research: analysis of consumer wrist-worn wearables. J Med Internet Res.

[2] Phillips SM, Cadmus-Bertram L, Rosenberg D, Buman MP, Lynch BM (2018). Wearable technology and physical activity in chronic disease: opportunities and challenges. Am J Prev Med.

[3] Shin G, Jarrahi MH, Fei Y, Karami A, Gafinowitz N, Byun A, Lu X (2019). Wearable activity trackers, accuracy, adoption, acceptance and health impact: a systematic literature review. J Biomed Inform.

[4] Wright SP, Hall Brown TS, Collier SR, Sandberg K (2017). How consumer physical activity monitors could transform human physiology research. Am J Physiol Regul Integr Comp Physiol.

[5] Henriksen A, Woldaregay AZ, Miroslav M, Gunnar H, Hopstock LA, Grimsgaard S, Replication data for Using Fitness Trackers and Smartwatches to Measure Physical Activity in Research. DataverseNO, 2021 10.18710/6ZWC9Z.

[6] Richardson S, Mackinnon D. Left to their own Devices? Privacy Implications of Wearable Technology in Canadian Workplaces. 2017.

[7] Richardson SM. Debra. Wearable Device Inventory, Queen's University. 2019; https://www.sscqueens.org/resources/wearable-device-inventory-2017. Accessed 2019 05–05.

[8] Vandrico. The wearables database. 2017; http://vandrico.com/wearables (No longer available). Accessed cited 2017 07–12.

[9] GsmArena. GsmArena. 2017; http://www.gsmarena.com/results.php3?sFormFactors=8. Accessed cited 2017 04–01.

[10] Wearables. Wearables.com. 2017. http://www.wearables.com/devices. Accessed 2017 07–12.

[11] Specbucket. Smart Watches. 2017; https://smartwatches.specbucket.com. Accessed 2017 07–12.

[12] Prisguiden. Prisguiden - Prissammenligning - Den enkleste starten på et godt kjøp. 2021; https://prisguiden.no. Accessed 2017 10–19.

